# Wildfire-Like Effect of a WhatsApp Campaign to Mobilize a Group of Predominantly Health Professionals With a University Degree on a Health Issue: Infodemiology Study

**DOI:** 10.2196/17051

**Published:** 2020-08-10

**Authors:** Vanja Kopilaš, Srećko Gajović

**Affiliations:** 1 Faculty of Croatian Studies University of Zagreb Zagreb Croatia; 2 Croatian Institute for Brain Research University of Zagreb School of Medicine Zagreb Croatia

**Keywords:** instant messaging, rumor, 5G mobile networks, participatory web, virality, infodemiology, infodemic

## Abstract

**Background:**

Online interactions within a closed WhatsApp group can influence the attitudes and behaviors of the users in relation to health issues.

**Objective:**

This study aimed to analyze the activity of the members of a WhatsApp group initiated to raise awareness of the possible health effects of 5G mobile networks and mobilize members to sign the related petition.

**Methods:**

We retrospectively analyzed data from the WhatsApp group of 205 members that was active during 4 consecutive days in August 2019. The messages exchanged were collected, anonymized, and analyzed according to their timing and content.

**Results:**

The WhatsApp group members were invited to the group from the administrator’s contacts; 91% (187/205) had a university degree, 68% (140/205) were medical professionals, and 24% (50/205) held academic positions. Approximately a quarter of the members (47/205, 23%) declared in their messages they signed the corresponding petition. The intense message exchange had wildfire-like features, and the majority of messages (126/133, 95%) were exchanged during the first 26 hours. Despite the viral activity and high rate of members openly declaring that they signed the petition, only 8 (8/133, 6%) messages from the group members, excluding the administrator, referred to the health issue, which was the topic of the group. No member expressed an opposite opinion to those presented by the administrator, and there was no debate in the form of exchanging opposite opinions.

**Conclusions:**

The wildfire-like activity of the WhatsApp group and open declaration of signing the petition as a result of the mobilization campaign were not accompanied by any form of a debate related to the corresponding health issue, although the group members were predominantly health professionals, with a quarter of holding academic positions.

## Introduction

The participatory web (Web 2.0) allows users to not only access and use the content but also create and exchange content during active interactions. Users are prosumers, in that they are both consumers and producers of the content [1]. When searching for knowledge, in particular for health-related knowledge, digital capabilities create a sophisticated environment described by a recently introduced metaphor – knowledge landscapes [2]. Knowledge is accessible (or hidden) in knowledge landscapes and can be approached by various individualized pathways shaped by personal, cultural, and societal contexts [3].

The participatory nature of the web also implies that online interactions influence attitudes, raise awareness, and mobilize users. With health, use of the digital environment, both intentional and unintentional, affects health-related behaviors and the health status of the interacting individuals [4]. The online environment immensely augments the number of participants, speed, and geographical reach of interactions. However, the dynamics and patterns of these interactions, which are at the core of the new digital society, are still mostly unknown. Subsequently, their health effects are not well understood and could go in both directions, either beneficial or harmful [5].

Here, we describe retrospective analyses of the content and temporal dynamics of a WhatsApp group that was created as a mobilizing campaign to raise awareness of the possible health effects of 5G mobile networks. The specific group topic (ie, the prospective effect of 5G mobile networks on human health or environment) will not be discussed here, as the topic was used just as a general paradigm of the incoming complex technology and health uncertainties due to its application.

Instant messaging services have become one of the most popular and commonly used communication tools [6]. Immediacy, privacy, and cost-free use are features that contribute to the popularity of instant messaging [6]. WhatsApp as a social media platform is a mobile-based instant messenger characterized by the exchange of messages in real-time, usually between two users or among a group of users. With over 1.5 billion users worldwide, WhatsApp has emerged as a leader in the instant messaging industry, outranking other services such as Facebook, WeChat, Viber, and Skype [6,7]. WhatsApp is already widely used for various health purposes, including health education [8], rapid consultations in surgery, obstetrics, or in case of stroke [9,10], and as a tool for support groups like smoking cessation or weight management [11,12]. Moreover, due to its private and controlled environment, WhatsApp has become an ideal platform for discussing current matters of interest spanning news, politics, and activism [6].

Since WhatsApp groups emerge spontaneously and are visible only to their members, they are mostly unavailable to be studied. The WhatsApp group described in this study was created, active, and completed prior to the conception of this project; therefore, the analyses presented here are retrospective. The health concerns and messages exchanged in the group created a profile of interactions, similar to the dynamics of disease (ie, epidemiology), but in the context of digital activity (ie, infodemiology) [13]. Subsequently, the presented WhatsApp group analyses provide new insight into health-relevant participatory digital activity.

## Methods

The initiator of the campaign, who undertook the role of the WhatsApp group administrator, invited individuals to the group via their mobile numbers. The WhatsApp group administrator was a medical doctor who, due to the nature of her or his profession and employment, was well connected with academics and medical professionals at local hospitals. The group was initiated at 2 pm on Saturday and had a total of 205 members. The group was active for 4 consecutive days (Saturday to Tuesday), after which it remained silent. Although 2 isolated messages were posted weeks later, they were not included in the analysis. The group was created during a 3-day weekend, as that Monday was a public holiday in Croatia. Moreover, August is regularly a period of vacations in Croatia. Thus, it is to be assumed that most of the group members were not at work until Tuesday (until the last day of the 4 consecutive days of group activity).

The idea to analyze the activity of the group was conceived after the group messaging had ceased. The WhatsApp group administrator was the only one who had knowledge of all members’ identities. The messages were collected and deidentified, and their content and timing were analyzed. The descriptive characteristics of the group members (eg, gender, profession, academic achievements) were obtained from the group administrator. We conducted a frequency analysis of the messages using the collected content and timing. All necessary precautions were taken to maintain member anonymity and avoid potential member recognition. Ethical approval for the study was obtained from the University of Zagreb School of Medicine.

External links in the messages were followed to verify their viability. Claims of forwarding the discussion to other social networks (ie, Facebook) were not verified. The daily number of petition signatures was collected from the corresponding petition website, but the identity of signatories was not matched to the WhatsApp group members due to their anonymity. The Google Trends analysis was performed for a 7-day period, to analyze searches performed in Croatia with the search term “5G.”

## Results

### Characteristics of the WhatsApp Group Members

The group consisted of 205 members (Table 1), with 187 members holding a university degree (187/205, 91%), and there were 140 health professionals (140/205, 68%), of which 125 were medical doctors (125/205, 61%) covering 16 different medical specializations. Furthermore, 75 (75/205, 37%) members had obtained a PhD, 50 (50/205, 24%) of whom held academic positions (professors and assistant professors). Although members were predominantly from the health sector, 8 members were electronic engineers and assumed to be educated to understand mobile communication technologies. All of the members were older than 18 years, and only 9 members were unemployed: 5 students and 4 retired individuals.

**Table 1:** Characteristics of the 205 members of the WhatsApp group.

Member characteristics		n (%)
**Gender**		
	Women	106 (52)
	Men	99 (48)
**Academic status**		
	Current student	5 (2)
	University degree	187 (91)
	PhD	75 (37)
**Profession**		
	Medical doctor	125 (61)
	Medical doctor with specialization	97 (47)
	Engineer	12 (6)
	Electronic engineer	8 (4)
	Retired	4 (2)
PhD with an academic position at a university		50 (24)

The WhatsApp group was initiated in August 2019 as a mobilizing campaign aimed to encourage members to sign a petition related to an incoming change to the city of Zagreb’s (Croatia) legislation regarding mobile network antennas. In the first message, the administrator described the selection criterion for members from her or his contacts as those who “do not think only about themselves, here and now” but also consider the “long-term wellbeing of our kids and our planet Earth.“ Together with the main aim, to sign the petition, it was clear upfront that the general aim was to raise awareness of the possible health effects of 5G mobile networks. The administrator immediately declared that the members were free to leave the group whenever they liked.

### Results of the WhatsApp Group

**Figure 1:**
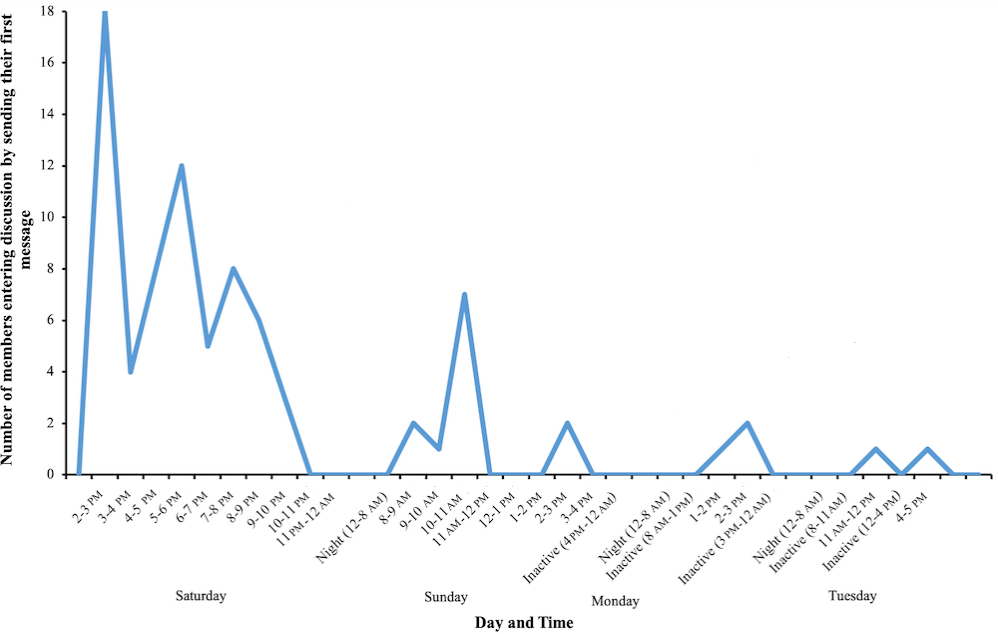
Number and time distribution of the members entering the discussion, as indicated by their first message.

Of the 205 group members, 81 (81/205, 40%) were active by posting messages (Figure 1, Table 2). From a total of 133 messages, 28 were generated by the group administrator, and 105 were generated by group members, which is 1.3 messages per active group member, not taking into account the administrator. This indicates that the group was active not only regarding the number of messages but also in particular by wide member participation.

**Table 2:** Timing and extent of the different features of WhatsApp group events by the 205 members.

WhatsApp group events		1 hour	4 hours	10 hours (up until the first night)	26 hours	Total (75 hours)
Members who posted their first message, n		18	42	64	76	81
**Messages posted, n**						
	All messages	30	72	103	126	133
	Administrator messages	8	17	21	26	28
Members who declared that they signed the petition, n		0	16	37	46	47
**Members who left the group, n**						
	Total	11	40	65	79	83
	Left the group after signing the petition	0	7	14	17	17
**Administrator responses to others, n**						
	Total	2	5	8	11	13
	Positive responses	2	5	8	11	13
	Negative responses	0	0	0	0	0
**Emojis used, n**						
	Total	10	26	35	40	49
	Positive feelings	9	24	33	35	44
	Negative feelings	1	2	2	5	5
**Messages that referred to 5G technology, n**						
	Total	5	12	14	19	19
	Rumors	2	7	8	12	12
	Not rumors	3	5	6	7	7

**Figure 2:**
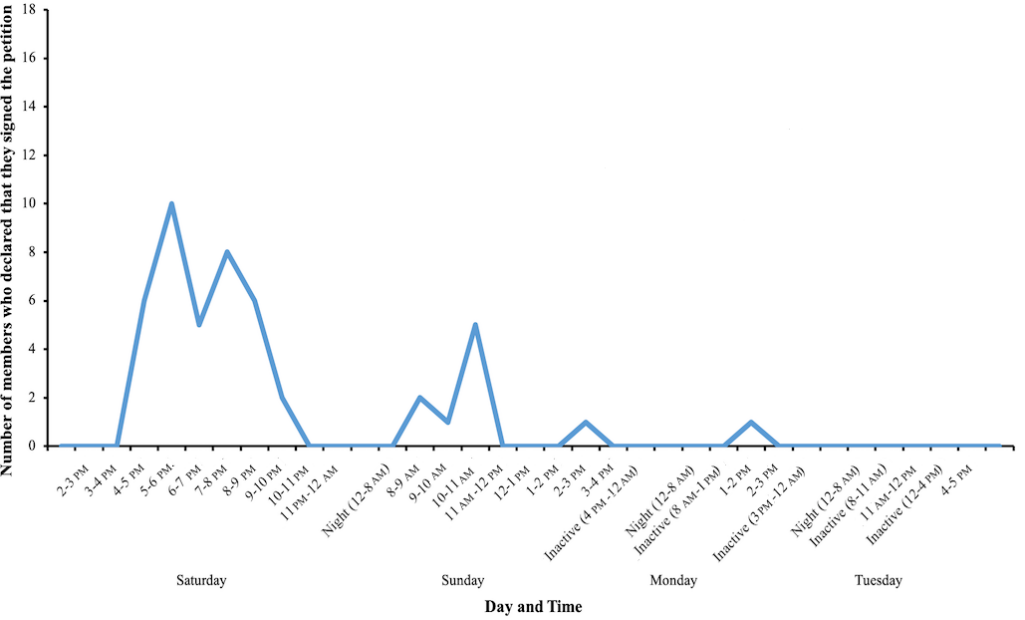
Number of members and the time distribution of their messages declaring they signed the petition.

A notable result of the WhatsApp group was open declaration of signing the petition, which was the clear aim of the mobilizing campaign. The members of the group influenced their peers to sign the petition by declaring to others in their messages that they had signed it (Figure 2). A total of 47 (47/205, 23%) members openly declared signing the petition in their messages. From the petition website, it was possible to get insight into the daily statistics of new signatures. The day before the onset of the WhatsApp group, only 2 signatures were collected, which changed abruptly to 50 on the first day after the formation of the WhatsApp group and 42 more on the second day of the group activities. For the 3 subsequent days, the number of signatures was still above average (15-19 signatures/day), and 6 days after, it dropped to 4 signatures and stayed at this level in the days that followed. There were 137 total signatures collected during these 5 days coinciding with the WhatsApp group activity. Due to the anonymous analysis of the group activities, it was not possible to connect the names of the signatories published at the petition site with the identities of the WhatsApp group members. Therefore, it was not possible to know exactly how many signatures, beyond the 47 signatures that were openly declared in the WhatsApp group messages, were the effect of the mobilizing campaign. The group members themselves declared in 7 messages that they were sharing the discussion outside the WhatsApp group, and in 3 messages, Facebook was explicitly named as the platform for sharing. Facebook was the only other social network mentioned in the conversation.

### Message Characteristics in the Group Activity

**Figure 3:**
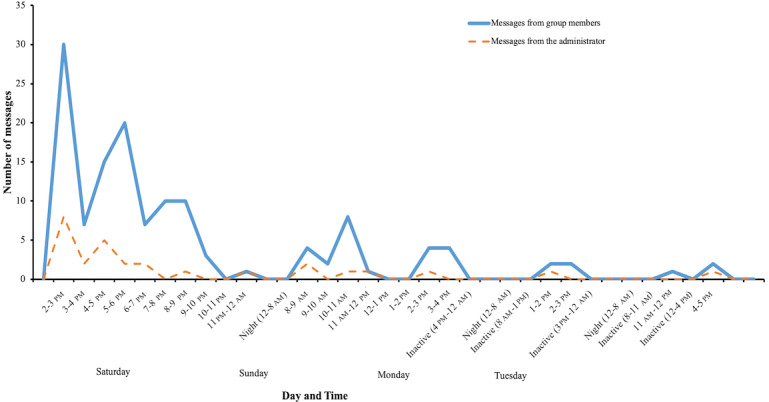
Number and time distribution of the messages from the members versus those from the administrator.

The actigraphy of the group showed that the total active time of the WhatsApp group was from Saturday 2 pm until 5 pm on Tuesday, or a total of 75 hours (Figure 3). This period included 4 periods of inactivity of 53 hours total (8 inactive hours Saturday night, 21 inactive hours Sunday night, 20 inactive hours Monday night, and 4 inactive hours during Tuesday daylight). This indicates that there were only 22 (29%) active hours from a total of 75 hours of continuous message exchanges.

Most of the activity was concentrated in the first 26 hours of group existence (from Saturday 2 pm to Sunday 4 pm), during which time, 126 messages were exchanged (95% of the 133 total messages), and 76 members posted to the group (94% of 81 active members; Figures 1 and 3). Only 7 messages were exchanged, and 5 members became active in the next 49 hours. The 26-hour period included 8 hours of nighttime, from midnight to 8 am, when the group was inactive. Subsequently, regarding active hours, the first period contained 18 active hours to generate 126 messages, and the next period had only 4 active hours to generate 7 messages, although it extended through 3 calendar days or 49 hours (Figure 3).

The maximum activity was reached already in the first hour of the group’s existence (30 messages, of which 8 were from the group administrator; 18 members posting messages; Figures 1 and 3). By the fourth hour of activity, 54% (72/133) of the total number of messages was reached, and during the same time, 52% (42/81) of active members posted their first message. The fourth hour was the second most active hour, with 20 messages exchanged. Before the night break (Saturday midnight, 10 hours of activity from the onset), 103 (103/133, 77%) messages were exchanged, and 64 (64/81, 79%) members produced a message. These data clearly showed that the onset of activity regarding both messaging and involvement of the group members was very rapid and concentrated at the very beginning of the activities.

### Communication Content and Extent of the Debate

**Figure 4:**
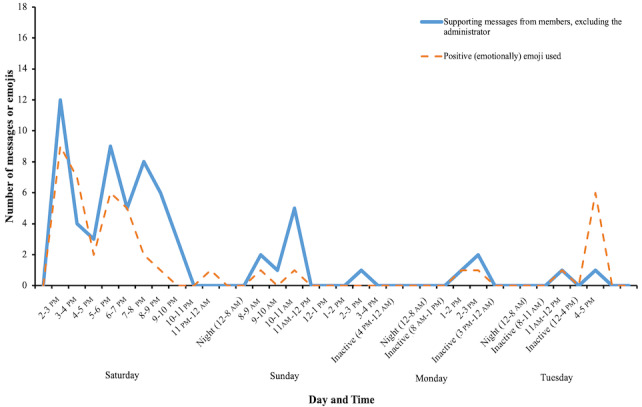
Number and time distribution of messages from the members supporting the administrator and of positive (emotionally) emojis posted.

The role of the administrator was rather pronounced. The administrator generated 28 messages (21% of the total 133 messages), stirring the discussion and responding to the members. The administrator’s responses to others were exclusively positive, confirming the group members’ statements or praising the members personally. The members supported the administrator in 64 messages (64/133, 61%), and in 29 messages (29/133, 28%), they directly complimented the administrator (Figure 4). The emotional aspects of the messages were reflected by the use of emojis (total of 49), where 44 emojis were used depicting positive emotions, compared to only 5 negative emojis (Figure 4).

It should be noted that the administrator never asked for a comment or a response from a specific member, but only responded to the messages posted. Moreover, the administrator refrained from attempting to restart the activities after inactive periods, and the activities were restarted by the members themselves. Therefore, the final cessation of activities could be considered as a spontaneous turning off of the members’ interest.

Despite the lively group activity, the specific topic of the WhatsApp group (ie, 5G mobile networks) was elaborated in only 19 (19/133, 14%) messages; of these, 11 were from the administrator. It should be emphasized that only 8 (8/133, 6%) messages from group members (excluding the administrator) referred to the specific topic of the group. This could be compared to the 7 messages posted, which were utterly unrelated to the exchanged WhatsApp group messages (eg, pictures of nature, mentioning excursions or bicycling together).

**Figure 5:**
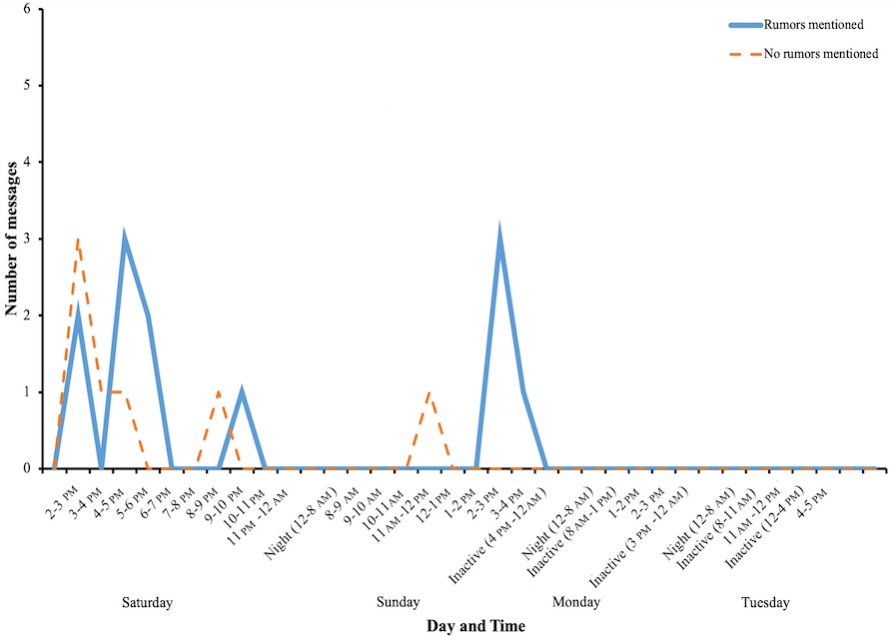
Number and time distribution of messages elaborating the group topic (influence of technology on health) that contained a rumor versus equivalent messages that did not contain a rumor.

In the 19 messages dealing with the topic of 5G mobile networks, 10 outside resources were referenced. These included 1 link to a published research article, 1 link to a YouTube video created by Croatian national television, 1 link to a magazine article, and 7 links to various web pages. All of the outside references supported the viewpoints of the administrator. From the 19 messages discussing the topic of the group, 12 messages mentioned a rumor related to the topic in contrast to only 7 messages without rumors (Figure 5).

No message stated a counterargument or opposed the viewpoints of the administrator. Subsequently, there was no real debate in the form of a series of opposing messages exchanged. Only 2 messages could be considered vaguely opposite to those declared by the administrator: a message claiming “Sorry, this is not important,” after which the member immediately left the group, and another message stating, “I consulted the friends from Faculty of Natural Sciences and Faculty of Engineering,” after which the member immediately left the group. The administrator repeatedly urged in 8 messages that members should undertake their research and form their own opinion about the topic.

**Figure 6:**
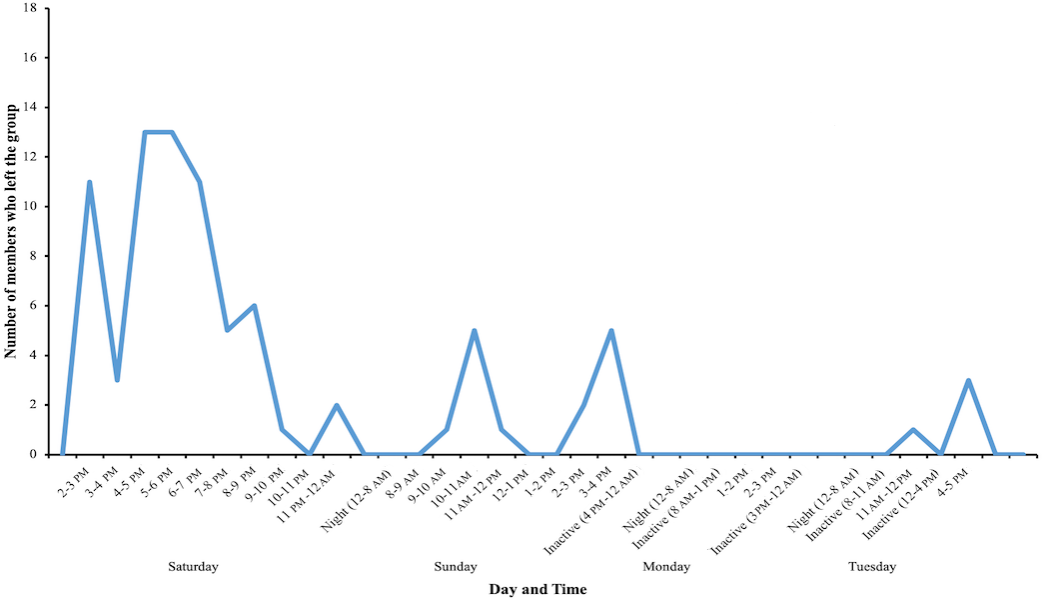
Number and time distribution of members who left the group.

During the whole period of the WhatsApp group activity, 83 (83/205, 40%) members left the group (Figure 6); 17 members left the group after openly declaring to have signed the petition, and 9 members left even before the administrator sent the first message explaining the purpose of the group. Subsequently, if we could speculate on the silent expression of disagreement from the group members, 124 (124/205, 60%) members never produced a message, and 57 (57/205, 28%) left the group without letting the others know the reason they left.

The Google Trends results for “5G” as a topic in Croatia for this period were inconsistent. The results retrieved for the same period (7 days) but including the day before or the day after showed remarkably different results in relation to the days of WhatsApp group activity. Subsequently, the only resource to estimate whether there were any related Google searches by the group members was not effective.

## Discussion

### Wildfire-Like Dynamic of the WhatsApp Group

The mobilizing campaign described here and executed through the creation of the WhatsApp group could be judged remarkably successful. The one measure of success was the fulfillment of the immediate aim of the campaign, signing the corresponding petition, which was openly declared by less than a quarter of the group members. The other measure of success was the involvement of the group members in lively digital activity (40% of the members), each posting 1.3 messages on average, one message per 2 minutes in the peak activity period. The fact that all members were acquaintances of the group administrator and had their telephone numbers in the administrator’s contact list, indicated that possibly similar effects could be achieved without the help of the digital environment by merely contacting the members individually. However, what clearly distinguished the digital from ”offline“ scenarios was the speed and intensity of the group activities (ie, the wildfire-like dynamic) [14].

The description of wildfire-like dynamics is based on the rapid initiation of the message exchanges (with the first hour being the most active), involvement of the invitees in the messaging process (40% of the members), short duration of the intense activity (50% of messages were posted within the first 4 hours, and 95% were posted within the first 26 hours), and subsequent rapid stopping of the activities (after 4 days). Despite its short and intensive lifespan, the group achieved the initial purpose of mobilizing the members to sign the petition.

It could be argued whether the term wildfire is used correctly here, as it denotes both rapid and wide geographical spread of the message, considering that the group had only 205 members. However, as the WhatsApp group is closed, the “forest” of the members was limited, and when ”burnt,“ the fire stopped. The digital wildfire was conceived originally as a term to depict the spread of rumors or malicious information causing significant harm [15]. Analyzing digital activity in the form of infodemiology has a similar connotation to the epidemiology of a disease, with diseases viewed negatively. We would like to argue against this normative approach to the phenomenon to be considered negative or bad *per se* (vs positive or good), as the same dynamics can be achieved regardless of the normative nature of the information. In this study, we intentionally avoided discussing the normative classification of the particular topic (ie, the relation between 5G mobile technologies and health). Whatever the topic, it deserves to be discussed in the digital environment of a social network. Considering that social networks represent a base for any grassroots movement, attaching a normative tag (bad vs good, harmful vs beneficial) could be at least controversial if not counterproductive in the sense of participatory democracy. Therefore, we claim here that wildfire-like dynamics are typical for the digital environment, as a specific feature of the digital society, and would happen in favorable circumstances regardless of the content, certainly not only if the content is malicious.

Another controversy related to using the term wildfire is its obvious overlap with the term virality, which refers to the viral-like spread of digital content. The observed dynamics visualized by the actigraphy in this study corresponds to viral event signatures [16]. Our choice to refer to the WhatsApp group activity as wildfire was based on the lack of specific content shared in the WhatsApp group being a “virus;” rather, the group shared a concern on a possible health issue more broadly. Subsequently, the term virality could be used to describe the spread of digital content, while wildfire could encompass overall mobilization and response of the users, which was initiated by viral content.

When discussing the circumstances contributing to the success of the mobilizing campaign and its wildfire-like attributes, four concepts could be considered: trust, motivation, situation, and narrative context [17]. All these concepts bring attention to the initiator and administrator of the WhatsApp group, who, in our opinion, should be praised for the group achievements. In the sense of “trust,” the group members all knew the administrator personally, and there was a substantial number of messages supporting the administrator (64% of the total 205 messages) or directly complimenting the administrator (28% of the total). “Motivation” was stirred by the administrator being the most active group member in posting messages (21% of the messages) and providing the arguments, including external links to their own material, to support the signing of the petition. The enhancing contributor was the “situation” of the vacation period and long weekend, providing less distraction from daily routines. Finally, the predominant “narrative context” was the appealing fight of the individuals versus the governing elite (the parliament of the city of Zagreb) and versus corporate interests (through the introduction of new lucrative technology). The posteriori analyses showed that in every dimension analyzed — trust, motivation, situation, and narrative context — the group had all prerequisites to acquire wildfire-like features.

The analysis of the WhatsApp group dynamic presented the features of emotional contagion as well, where emotions were shared among the interacting netizens in the digital environment, influencing the spread of information and attitudes [18,19]. The emotions were specifically shared through emojis, which were abundantly used in composing the message (37% of the messages), showing predominantly positive emotions [20]. Interestingly, the dynamics of this study’s WhatsApp group can be compared to the recently published emotional contagion simulation on rumor refuting, which indicates that after initial input, the group will stabilize in 4 days (same as in the group in this study), and that none of the subjects would act negatively to what has happened but rather positively (agreeing with the initial input) or neutrally (indifferent to the topic) [21]. Moreover, the success of the current WhatsApp group can be related to the fact that most of the members were naïve both to the digital platform and to the topic, while subsequent attempts for mobilization on some other topic can fail due to the cry-wolf phenomenon or neutral status of the members [22].

### Absence of Debate

The analysis of the WhatsApp group dynamics indicated that its dynamics can be related to the previously suggested mechanisms of rumor distribution and emotional contagion. However, these mechanisms, although applicable to the analyzed WhatsApp group, are not relevant to the topic of discussion. Subsequently, we were rather keen to identify the comments that elaborated the specific topic of the group (ie, 5G mobile communications and health). The advantage of the group was that its members were predominantly health professionals with university degrees, and many held academic positions. Therefore, it is to be assumed that the members were educated and had above-average capabilities to grasp complex health-related and technology-related issues. However, although the dynamic of the messaging had a wildfire-like feature and some group members responded to the mobilizing campaign by signing the petition, the posted messages elaborating the topic of the group were surprisingly rare. What was surprising is that none of the messages opposed the administrator, and no sequence of messages was exchanged expressing opposing opinions that would represent the presence of a digital debate. The ambiguous sign of disapproval could be inferred by members leaving the group or “lurkers” not posting anything; however, they certainly did not act in the sense of debating the issue.

The absence of opposing opinions and debate is rather alarming, as we assume that the digital environment is an ideal forum for the exchange of divergent opinions and a place where an eventual social dispute could be settled [23]. As the digital environment poses no barriers, the societally relevant controversies and subsequent reconciliations are open to everyone interested. The digital capabilities allow the relevant discussions to be amplified and involve a wide range of participants (ie, total humanity in the idealistic sense). Subsequently, digital technology could serve as a basis for the all-embracing participatory democracy [24].

However, in the current example, this entire concept was missing, although the participants, according to their abilities (university education and academic positions) and professions (health and information technology professionals), were well suited to engage in a debate. The topic of the current study is rather specific to claim whether this was an exception or a rule, as we lacked the eventual comparison with similar situations; this is a major limitation of this study.

Several elements may indicate reasons why the debate was not present. First, the exchange of the messages was rather hierarchical, from the administrator toward the members and back to the administrator, with no messaging among the members. Subsequently, any opposing opinion would be a direct confrontation to the administrator, challenging the hierarchy and not just a dispute between some of the members. None of the members gained an “extra force” (as opinion leader, motivator, or central node in the network) during the message exchange. Not to be forgotten is that all members were the administrators’ contacts in the offline world. Additionally, the wildfire-like effect of fast message exchanges left less time for individual research on the topic (despite the fact that the administrator urged the members to form their own opinion through their approach). Finally, the timing of the activities was during the weekend and vacation period, and we assume that most of the participants were at home or out of town, leaving them in a position to be unable to meet each other. The assumption that nonvirtual real-life conversation is needed for the consolidation of opinion, regardless of online virtual influences, is a topic worth exploring in the future.

### Conclusions

In conclusion, the WhatsApp group had wildfire-like features and served to mobilize the members to act publicly in relation to the health issue. However, the very topic of controversy (effects of 5G mobile technologies on health) was not challenged in the form of debate, although the group members were predominantly health professionals and held academic positions.
